# Correction: Groffmann et al. Identification of a New and Effective Marker Combination for a Standardized and Automated Bin-Based Basophil Activation Test (BAT) Analysis. *Diagnostics* 2024, *14*, 1959

**DOI:** 10.3390/diagnostics15202571

**Published:** 2025-10-13

**Authors:** Johannes Groffmann, Ines Hoppe, Wail Abbas Nasser Ahmed, Yen Hoang, Stefanie Gryzik, Andreas Radbruch, Margitta Worm, Kirsten Beyer, Ria Baumgrass

**Affiliations:** 1 German Rheumatology Research Center (DRFZ), A Leibniz Institute, 10117 Berlin, Germany; 2 Department of Rheumatology and Clinical Immunology, Charité—Universitätsmedizin Berlin, 10117 Berlin, Germany; 3 Division of Allergy and Immunology, Department of Dermatology, Venerology and Allergy, Charité—Universitätsmedizin Berlin, 10117 Berlin, Germany; 4 Department of Pediatric Respiratory Medicine, Immunology and Critical Care Medicine, Charité—Universitätsmedizin Berlin, 13353 Berlin, Germany; 5 Institute of Biochemistry and Biology, Faculty of Science, University of Potsdam, 14476 Potsdam, Germany

There was an error in the original publication [1] regarding the antibody concentration.

A correction has been made to Materials and Methods, Basophil Activation Test, Paragraph 1:

Fresh heparinized whole blood was mixed with RPMI 1640 Medium, GlutaMAX™ (Thermo Fisher Scientific, Waltham, MA, USA) with a ratio of 1:1 or 5:2, preheated to 37 °C, containing 6.25 µg/mL CD63 antibody and the allergen extract or control.

Another error in the original publication concerned the spelling of the cited author’s name, “Behrends”.

A correction has been made to Discussion, Paragraph 4:

So far, there are two remarkable automation approaches by Patil et al. [34] and Behrends et al. [25] that both utilize automated gating strategies using Bioconductor tools in the R environment.

The authors state that the scientific conclusions are unaffected. This correction was approved by the Academic Editor. The original publication has also been updated.
