# Supplementary material for: Identification of a New and Effective Marker Combination for a Standardized and Automated Bin-Based Basophil Activation Test (BAT) Analysis
Source: Diagnostics (Basel). 2024 Sep 4;14(17):1959. doi: 10.3390/diagnostics14171959 (PMC11394212; doi:10.3390/diagnostics14171959)
Supplement: Supplementary file 1 [file diagnostics-14-01959-s001.zip › Supplementary_Materials_Ver2.pdf]

Supplementary Materials

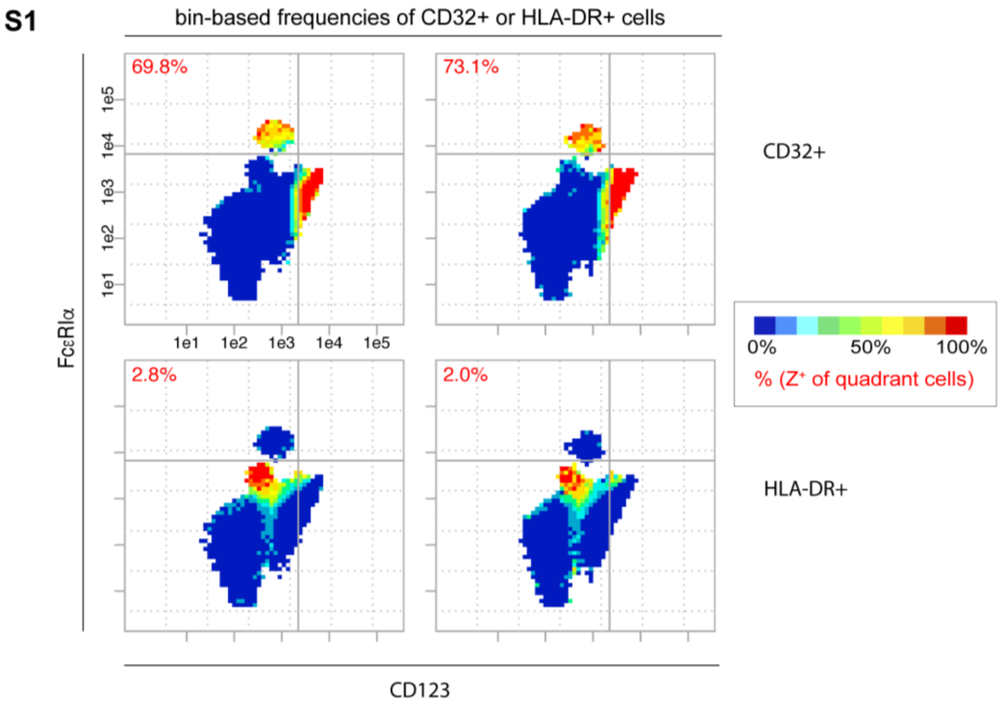

Supplementary Figure S1. Frequency bin-plots of unstimulated samples with basophils as CD123^{low} and FcεRIα^{high} on the x- and y-axis separated in the upper left quadrant. CD32 (top row) and HLA-DR (bottom row) as auxiliary basophil identification marker on the z-axis. Bin-colors reflect the frequency in percent of respective z-marker^{+} cells within a bin.

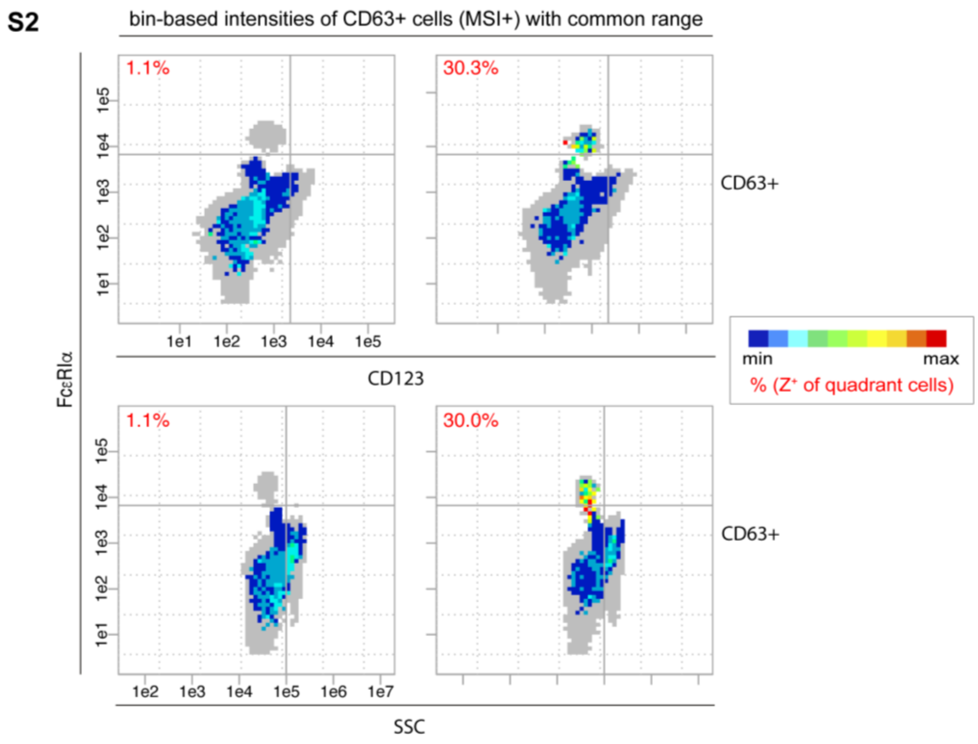

Supplementary Figure S2. MSI+ bin-plots of unstimulated samples (left) and anti-IgE stimulated samples with basophils as CD123^{low} (top row) or SSC^{low} (bottom row) and FcεRIα^{high} on the x- and y-axis separated in the upper left quadrant. CD63 is used as basophil activation marker on the z-axis. Bin-colors reflect the mean signal intensity of cells per bin that are positive for CD63.

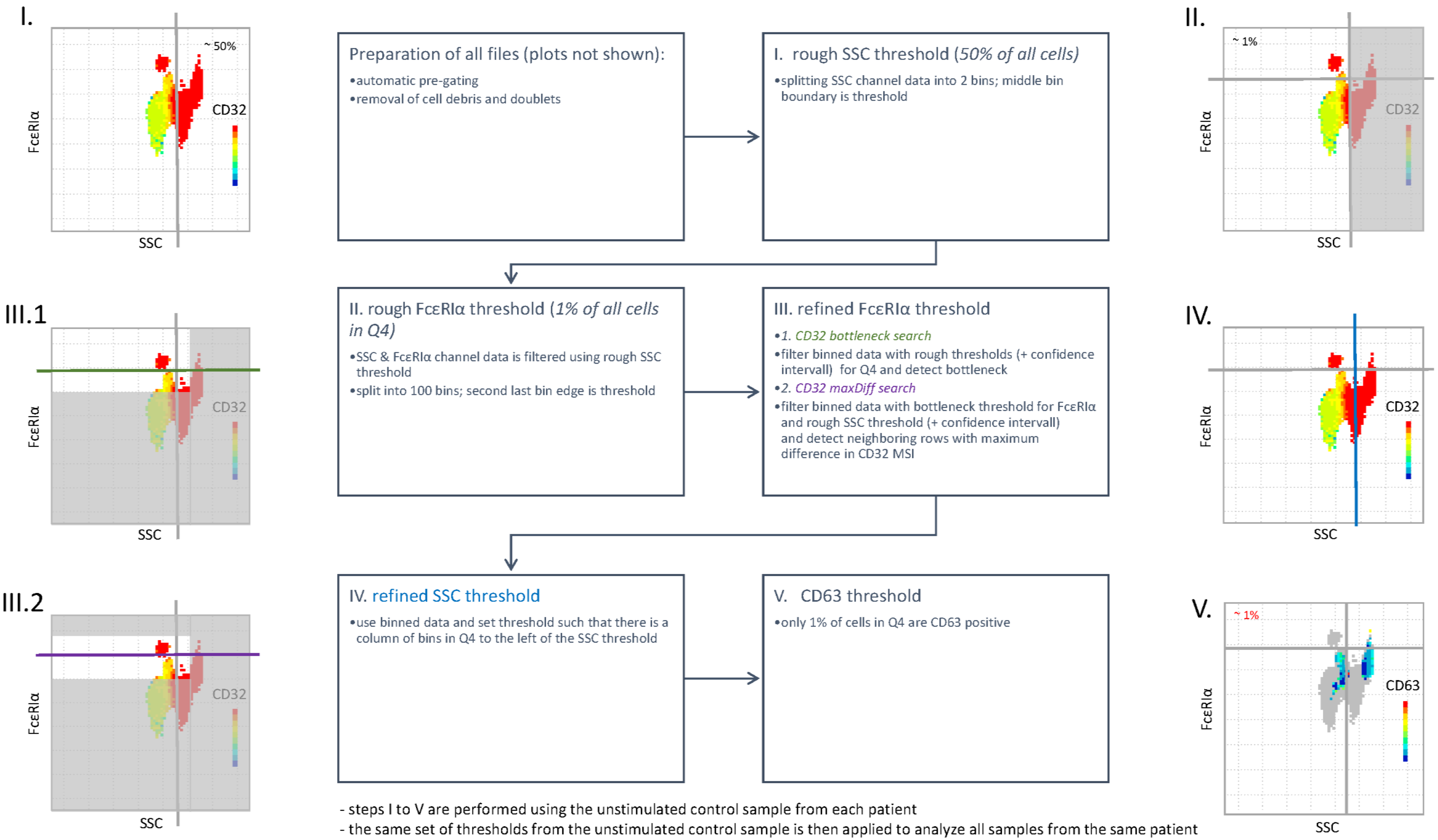

Supplementary Figure S3. Graphical description of bin-based auto-BAT procedure.

The auto-BAT workflow utilizes an automated pregating process followed by multiple steps to refine thresholds, ensuring accurate identification and isolation of basophils. In the following text, the Roman numerals are used to reference the particular steps in the supplementary Figure 3.

The thresholding process is preceded by pregating. In this step, all files are pregated on single cells, excluding doublets, thrombocytes, and cell debris. Subsequently, thresholding begins on the unstimulated sample: Initially, the SSC channel data is divided into two bins, with the middle bin boundary serving as the threshold. This threshold is set such that 50% of all cells fall into quadrants Q2 and Q3 **(I)**. Using this coarse SSC threshold, the data is then filtered and further divided into 100 bins. The second last bin edge sets the coarse FcεRIα threshold, aiming for 1% of all cells in Q4 **(II)**.

The refinement of the FcεRIα threshold is based on typical patterns of the marker combination in the binned data. There is often a bottleneck separating the basophils from other populations. In addition, there are abrupt and strong changes in the intensity of the CD32 staining separating the basophiles. Technically, to refine the FcεRIα threshold, the binned data of the unstimulated sample is analyzed using the coarse thresholds in order to detect the bottleneck in CD32 expression, which indicates the basophils **(III.1)**. A search for the maximum difference in CD32 is then performed using the bottleneck threshold and the coarse SSC threshold to constrain the search area. The row with the maximum difference in CD32 intensity is identified to finalize the FcεRIα threshold **(III.2)**.

Subsequently, the SSC threshold is refined using the binned data to ensure clear separation of basophils from other populations. The SSC-A threshold is set so that there is a column of bins in Q4 to the left of this threshold **(IV)**. Finally, the CD63 threshold is set to ensure that, in the unstimulated sample, only 1% of cells in Q4 are CD63 positive **(V)**.

These refined thresholds and multi-parameter binning are then applied to all BAT and donor samples to generate plots and calculate key metrics, ensuring a robust and reliable analysis.

The following criteria generate warning symbols and messages in the auto-BAT results for intuitive assessment of analysis quality and optional manual corrections, thereby reducing potential errors.

- The total number of events after pregating is below 100000
- The total number of cells in the upper left quadrant is below 350
- The total number of colored bins in the unstimulated sample is below 31, one bin contains ≥ 10 CD63+ cells
- The threshold setting for SSC did not function with the default parameters, leading to the activation of implemented drop back options.
- CD63 threshold above 10.5 or below 4.0
